# Meta-analysis of bariatric surgery versus non-surgical treatment for type 2 diabetes mellitus

**DOI:** 10.18632/oncotarget.11961

**Published:** 2016-09-10

**Authors:** Guo-zhong Wu, Bing Cai, Feng Yu, Zheng Fang, Xing-li Fu, Hai-sen Zhou, Wen Zhang, Zhi-qiang Tian

**Affiliations:** ^1^ Department of General Surgery, The 101st Hospital of Chinese PLA, Wuxi, Jiangsu, China; ^2^ Department of General Surgery, Wuxi People’s Hospital, Nanjing Medical University, Wuxi, Jiangsu, China; ^3^ Health Science Center, Jiangsu University, Zhenjiang, Jiangsu, China; ^4^ Nanjing Lishui People’s Hospital, Nanjing, Jiangsu, China

**Keywords:** bariatric surgery, type 2 diabetes mellitus, non-surgical treatment, meta-analysis

## Abstract

**Background:**

To compare short-term and long-term results of bariatric surgery vs non-surgical treatment for type 2 diabetes mellitus (T2DM).

**Methods:**

A systematic search was conducted in the PubMed, Embase, and Cochrane Library databases for randomized controlled trials (RCTs). All statistical analysis was performed using Review Manager version 5.3. The dichotomous data was calculated using risk ratio (RR) and continuous data was using mean differences (MD) along with 95% confidence intervals (CI).

**Results:**

A total of 8 RCTs with 619 T2DM patients were analyzed. Compared with non-surgical treatment group, bariatric surgery group was associated with higher rate T2DM remission (RR = 5.76, 95%CI:3.15-10.55, *P* < 0.00001), more reduction HbA_1C_ (MD = 1.29, 95%CI: -1.70 to -0.87, *P* < 0.00001), more decrease fasting plasma glucose (MD = -36.38, 95%CI: -51.76 to -21.01, *P* < 0.00001), greater loss body weight (MD = -16.93, 95%CI: 19.78 to -14.08, *P* < 0.00001), more reduction body mass index (MD = -5.80, 95%CI: -6.95 to -4.64, *P* < 0.00001), more decrease triglyceride concentrations (MD = -51.27, 95%CI: -74.13 to -28.41, *P* < 0.0001), and higher increase density lipoprotein cholesterol (MD = 9.10, 95%CI: 7.99 to 10.21; *P* < 0.00001). But total and low density lipoprotein cholesterol were no significant changes.

**Conclusion:**

Bariatric surgery for T2DM is efficacious and improves short- and long-term outcomes as compared with non-surgical treatment.

## INTRODUCTION

Type 2 diabetes mellitus (T2DM) is a disease with high prevalence, associated with severe co-morbidities as well as being a huge burden on public health [1]. Strict glycemic control is known to decreases long-term morbidity and mortality [2]. The current standard therapy for T2DM is medical treatment which cornerstone is intensive lifestyle modification strategies [3]. However, only partially patients achieve adequate glycemic control and a reduction in cardiovascular risk [4–6].

Bariatric surgery has recently emerged as new therapeutic approach for patients with T2DM. Several observational studies [7–10] and randomized controlled trials(RCTs) [11–17] have shown T2DM remission after bariatric surgery [18]. But until recently, it is not fully understood the etiology of diabetes remission following bariatric surgery. Also, these trials have some limitations, such as a small sample size of the various types of surgery, uncertainty the durability of the metabolic benefits of surgery, long-term safety, quality of life, and effects on diabetes-related end-organ disease. These studies could inevitably increase the risk of bias being responsible for their conclusions, and more strong evidences are still needed to support the results of these studies.

The meta-analysis can test hypotheses about sources of differences and assess the magnitudes of biases when used to compare results from different studies. So, we performed a meta-analysis to obtain comprehensive estimates of the clinical benefit from all of the available data. This meta-analysis identified and screened the short-term and long-term results of bariatric surgery compared with non-surgical treatment for T2DM in RCT studies.

## RESULTS

### Search results

**Figure 1:**
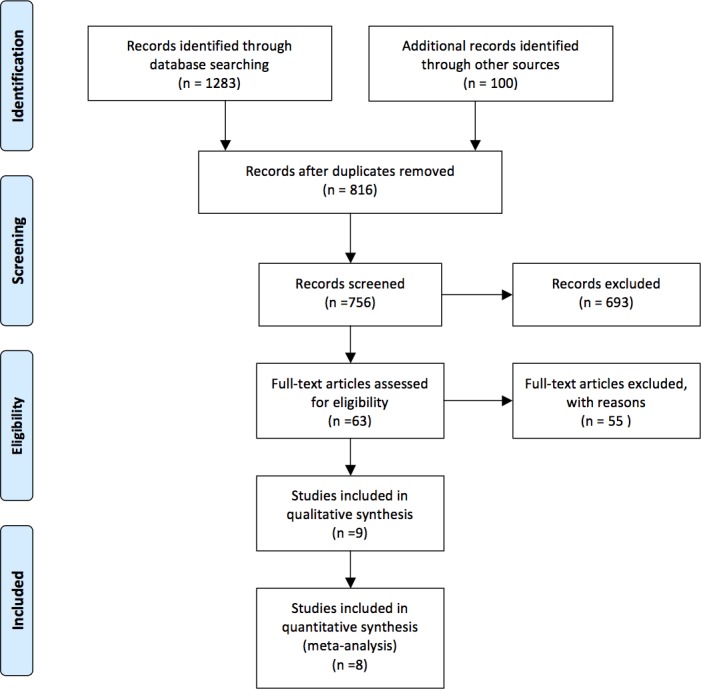
Flow diagram showing selection of relevant articles in this meta-analysis

A total of 816 articles were identified in a combined search of the electronic databases covering studies published before June 15^th^ 2016. 693 studies were in total excluded after title/abstract screening, then 55 studies were excluded following a further review full-text of the remaining 63 studies. In the end, 8 studies [12, 19–25] were included in this meta-analysis. Figure 1 demonstrated a flow chart of the selection process.

All of 8 studies were RCTs and conducted in Australia (1), China (1), Italy (1), and US (5). The majority of studies reported data from a single center and only one study [21] was multi-center study. Almost all studies did not report the numbers of participating surgeons and noted that patients were consecutively enrolled. 3 studies [21, 22, 24] had sample sizes greater than 100 patients. Two studies [20, 22] had 1-year follow-up, three studies [12, 21, 25] had 2-year follow-up, two studies [19, 24] had 3-year follow-up, and only one study [23] had 5-year follow-up. The characteristics of these 8 studies were described in Table 1.

**Table 1:** Basic characteristics of 8 studies included in this meta-analysis

Study (Author, year)	Country^a^	Design	Bariatric Surgery	Conventional Medical Treatment	T2DM variables,	IRB, IC, Reg	Follow-up (year)
Courcoulas *et al.*, 2015 [19]	US	RCT, nonblinded	RYGB LAGB	An intensive lifestyle weight loss intervention	FPG, HbA_1C_	IRB, IC, Reg.NCT01047735	3
Dixon et al., 2008 [12]	US	RCT, nonblinded	LAGB	Conventional dietary and ifestyle change	FPG, HbA_1C_, insulin, HOMA IR	IRB, IC, Reg. ACTRN 012605000159651	2
Halperin et al. 2014 [20]	US	RCT, nonblinded	RYGB	An intensive diabetes medical and weight management	FPG, HbA_1c_	IRB, IC, Reg. NCT01073020	1
Ikramuddin et al., 2015 [21]	US	RCT, nonblinded, Multi-center trial	RYGB	Lifestyle and medical management	FPG, HbA_1C_	IRB, IC Reg.NCT00641251	2
Liang et al., 2013 [22]	China	RCT, nonblinded	RYGB	Usual care plus Exenatide treatment	FPG, HbA_1C_, insulin, HOMA IR	IRB, IC Reg.NCT01435980	1
Mingrone et al., 2015 [23]	Italy	RCT, nonblinded	RYGB BPD	Diet, exercise, lifestyle modification program	FPG, HbA_1C_	IRB, IC Reg.NCT00888836	5
Schauer et al., 2014 [24]	US	RCT, nonblinded	RYGB LSG	Intensive medical therapy	FPG, HbA_1C_	IRB, IC Reg. NCT00432809	3
Wentworth et al., 2014	Australia	RCT, nonblinded	LAGB	multidisciplinary diabetes care	FPG, HbA_1C_ HOMA IR	IRB, IC, ACTRN12609 000286246	2

^a^ Country of first author's affiliation. RCT, randomized controlled trial; RYGB, Roux-en-Y gastric bypass; LAGB, laparoscopic adjustable gastric banding; BPD, biliopancreatic diversion; LSG, laparoscopic sleeve gastrectomy; FPG, fasting plasma glucose; HbA1C, hemoglobin A1C; HOMA IR, insulin resistance by homeostatic model assessment; IRB, institutional review and/or ethics board approval obtained; IC, informed consent obtained; Reg., clinical trial registration number or governmental grant number;

A total of 619 T2DM patients were included in the meta-analysis: 341 in bariatric surgery group and 278 in non-surgery treatment group. Bariatric surgery techniques included Roux-en-Y gastric bypass, sleeve gastrectomy, laparoscopic adjustable gastric banding, and biliopancreatic diversion. Non-surgical treatment generally comprised alterations in dietary intake, physical activity, behavioral or lifestyle modification, and pharmacotherapy. Table 2 summarized the description of patients at baseline from these 8 studies.

**Table 2:** Description of patients at baseline from the 8 studies included in the meta-analysis

Study (Author, year)	Group	No. of patients	Age Mean(SD)	Female (%)	Type 2 diabetes Duration (y)	BMI Mean(SD)	Body weight(kg) Mean(SD)
Courcoulas *et al.*, 2015	Bariatric surgery (RYGB+ LAGB) *	41	46.6(7.3)	81	6.8(4.5)	35.6(3.0)	101.1(14.0)
	Control	20	48.9(4.7)	85	5.7(5.6)	35.7(3.3)	99.3(13.4)
Dixon et al., 2008	Bariatric surgery (LAGB)	30	46.6(7.4)	50	<2	37(2.7)	105.6(13.8)
	Control	30	47.1(8.7)	50	<2	37.2(2.5)	105.9(14.2)
Halperin et al., 2014	Bariatric surgery (RYGB)	19	50.7(7.6)	68	10.6(6.6)	36.0(3.5)	104.6(15.5)
	Control	19	52.6(4.3)	53	10.2(6.1)	36.5(3.4)	102.7(17.0)
Ikramuddin et al., 2015	Bariatric surgery (RYGB)	60	49(9)	63	8.9(6.1)	34.9(3.0)	98.8(14.0)
	Control	59	49(8)	57	9.1(5.7)	34.3(3.1)	97.9(17.0)
Liang et al., 2013	Bariatric surgery (RYGB)	31	50.8(5.4)	29	7.4(1.7)	30.5(0.9)	82.0(3.5)
	Control (control+control plus exenatide) *	70	51.4(6.2)	31	7.2(1.7)	30.3(1.7)	81.5(4.3)
Mingrone et al., 2015	Bariatric surgery (RYGB+BPD) *	38	43.3(7.8)	55	6	45.0(6.5)	133.9(26.8)
	Control	15	43.5(7.3)	50	6	45.1(7.8)	136.4(21.9)
Schauer et al., 2014	Bariatric surgery (RYGB+LSG) *	97	47.9(8.2)	68	8.2(4.9)	36.6(3.7)	103.7(16.0)
	Control	40	50.3(7.5)	68	8.8(5.4)	36.4(3.0)	104.5(14.2)
Wentworth et al., 2014	Bariatric surgery (LAGB)	25	53(6)	76	2.2(1.7)	29(1)	81(10)
	Control	26	53(7)	65	2.8(1.8)	29(1)	83(12)

RYGB, Roux-en-Y gastric bypass; LAGB, laparoscopic adjustable gastric banding; BPD, biliopancreatic diversion; LSG, laparoscopic sleeve gastrectomy; BMI, body mass index. *In three-arm studies, Courcoulas 2015, Mingrone 2015 and Schauer 2014 the two bariatric surgery groups were combined, and in Liang 2013 the two non-surgical groups were combined.

### Quality assessment

**Figure 2:**
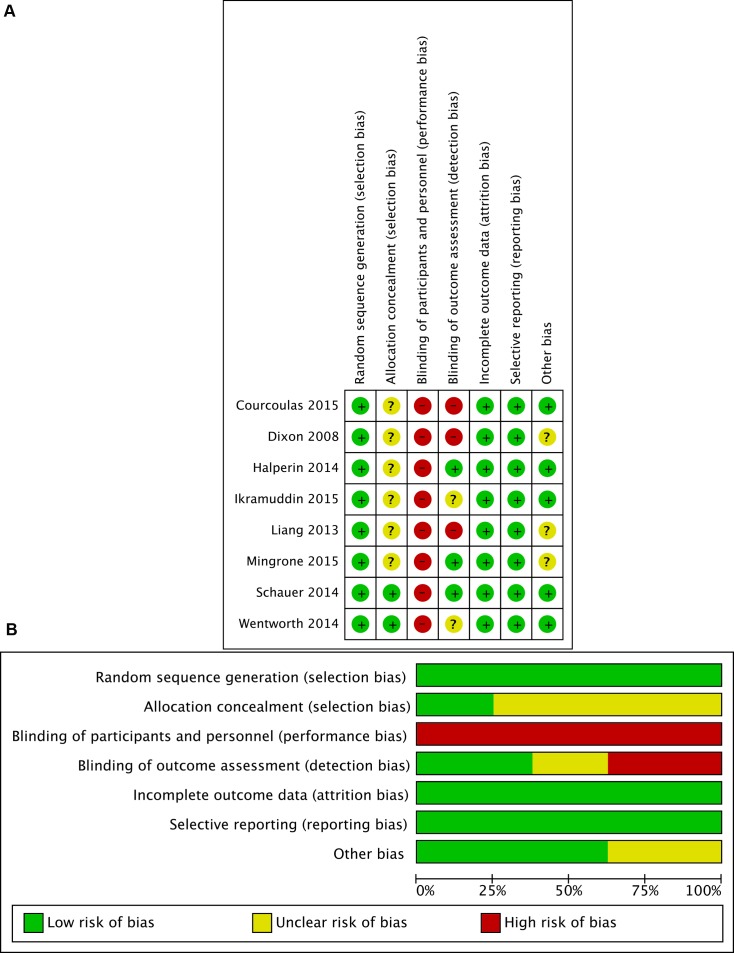
Assessment of risk of bias in this meta-analysis **A.** Summary of risk of bias for each study assessed by Cochrane Collaboration's tool **B.** Risk of bias graph about each risk of bias item presented as percentages across all included studies.

The risk of bias for each study was performed with Cochrane Collaboration's tool [26] (Figure 2A). All studies [12, 19–25] were adequate randomly generated the sequence. Six studies [12, 19–23] were unclear in clinic random allocation concealment. In all studies [12, 19–25], the participants and healthcare provider were not blinded. There were not the risk of incomplete outcome data and selective reporting in all studies [12, 19–25]. Therefore, there was a low risk of bias in this meta-analysis (Figure 2B).

### Meta-analysis of short-term outcomes

With respect to short-term outcomes, four endpoints including glycemic control (HbA_1c_, fasting plasma glucose (FPG)), body weight (weight loss, body mass index(BMI)), plasma cholesterol (low density lipoprotein(LDL), high density lipoprotein(HDL), total cholesterol), and triglyceride concentration were taken into analysis. Risk ratio (RR) along with 95% confidence intervals (CI) was calculated for dichotomous data, and mean difference (MD) with 95% CI was calculated for continuous data.

### Glycemic control

**Figure 3:**
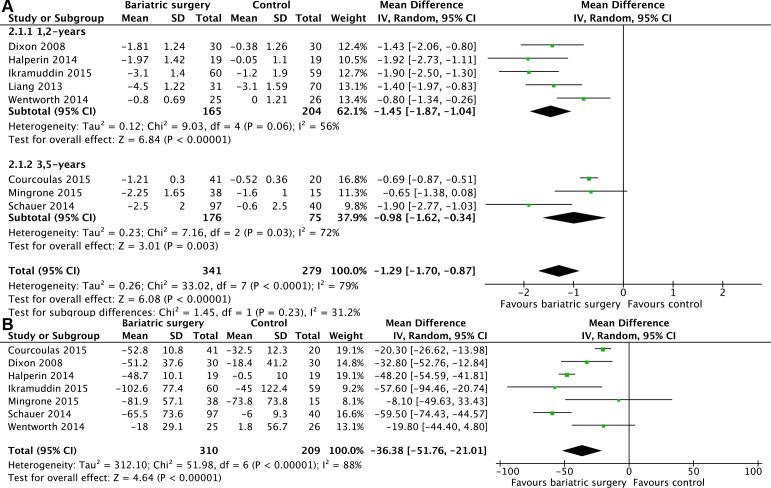
Mean changes in glycemic control after bariatric surgery versus non-surgical treatment (control) for Type 2 diabetes mellitus **A.** HbA1c levels (% points) **B.** fasting plasma glucose (mg/dL) (A conversion factor of 18 was used to convert values from mmol/L to mg/dL.).

All of 8 studies [12, 19–25] reported the mean changes in glycated haemoglobin HbA_1c_ level (%). Heterogeneity was high (*P* < 0.0001, *I*^2^ = 79%) and a random effect model was used. Bariatric surgery acquired more decrease of HbA_1c_ compared with non-surgical treatment for T2DM (MD = -1.29, 95%CI: -1.70 to -0.87, *P* < 0.00001) (Figure 3A). Further subgroup analysis showed that HbA_1c_ level reduction was not significantly different between the 1,2-year follow-up studies and the 3,5-year follow-up studies (difference of the mean difference 0.47%, *P* = 0.23). Statistical heterogeneity was moderate of the subgroup analyses (Figure 3A).

There were 7 studies [12, 19–21, 23–25] reported the mean changes in FPG level (mg/dL). Heterogeneity was high (*P* < 0.00001, *I*^2^ = 88%) and a random effect model was used. Bariatric surgery acquired more decrease of FPG compared with non-surgical treatment for T2DM (MD = -36.38, 95%CI: -51.76 to -21.01, *P* < 0.00001) (Figure 3B).

### Body weight

**Figure 4:**
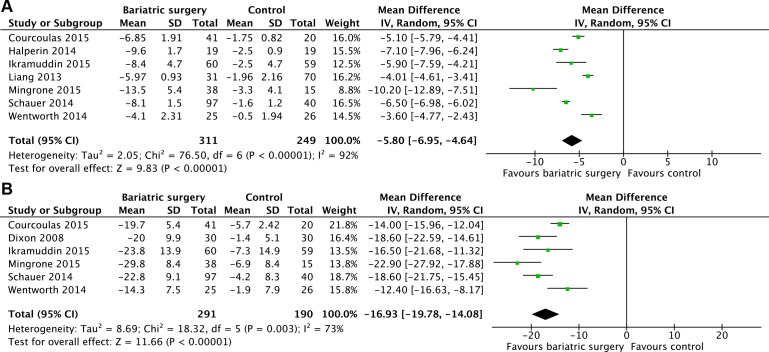
Mean changes in body weight after bariatric surgery versus non-surgical treatment (control) for Type 2 diabetes mellitus **A.** body mass index (kg/m2) **B.** percent weight loss (%).

7 studies [19–25] reported the mean changes of BMI (kg/m^2^). The results of the heterogeneity were *P* < 0.00001/*I*^2^ = 92% and indicated high heterogeneity among the studies. So a random effect model was used. Bariatric surgery acquired more reduction of BMI compared with non-surgical treatment for T2DM (MD = -5.80, 95%CI: -6.95 to -4.64, *P* < 0.00001) (Figure 4A).

6 studies [12, 19, 21, 23–25] reported the mean changes of body weight loss (%). Heterogeneity was high (*P* = 0.003, *I*^2^ = 73%) and a random effect model was used. Bariatric surgery acquired greater weight loss compared with non-surgical treatment for T2DM (MD = -16.93, 95%CI: -19.78 to -14.08, *P* < 0.00001) (Figure 4B).

### Plasma cholesterol

**Figure 5:**
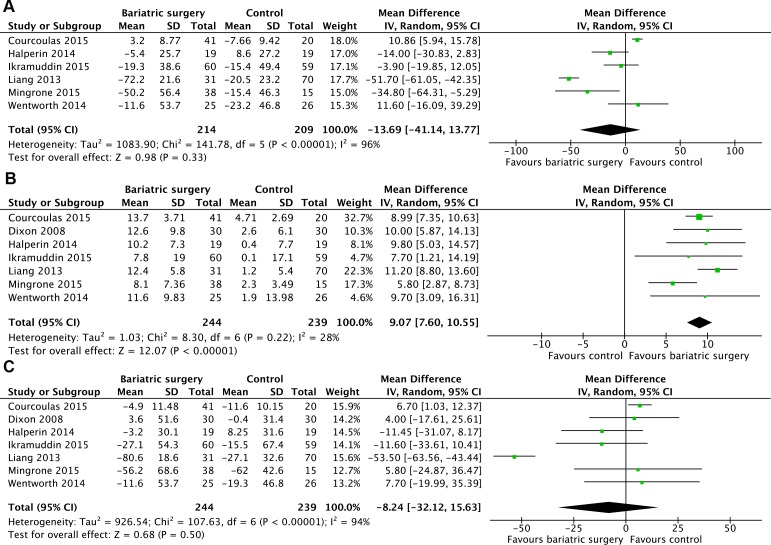
Mean changes in cholesterol concentrations (mg/dL) after bariatric surgery versus non-surgical treatment (control) for Type 2 diabetes mellitus A. low density lipoprotein cholesterol B. high density lipoprotein cholesterol C. total cholesterol (A conversion factor of 38.67 was used to convert values from mmol/L to mg/dL).

There were 6 studies [19–21, 23, 25] reported the mean changes in LDL cholesterol concentration (mg/dL). Heterogeneity was high among the trials (*P* < 0.00001, *I*^2^ = 96%) and a random effect model was used. Change of LDL cholesterol was no significantly different between the two groups (MD = -13.69, 95%CI: -41.14 to 13.77, *P* = 0.33) (Figure 5A).

There were 7 studies [12, 19–23, 25] reported the mean changes of HDL cholesterol concentration (mg/dL). Heterogeneity was low (*P* = 0.22, *I*^2^ = 28%) and a fixed effect model was used. Bariatric surgery acquired higher increase of HDL cholesterol compared with non-surgical treatment for T2DM (MD = 9.07, 95%CI: 7.60 to 10.55, *P* < 0.00001) (Figure 5B).

There were 7 studies [12, 19–23, 25] reported the mean changes of total cholesterol concentration (mg/dL). Heterogeneity was high (*P* < 0.00001, *I*^2^ = 94%) and a random effect model was used. Changes of total cholesterol was no significantly different between the two groups (WMD = -8.24, 95%CI: -32.12 to 15.63, *P* = 0.50) (Figure 5C).

### Triglyceride concentration

**Figure 6:**
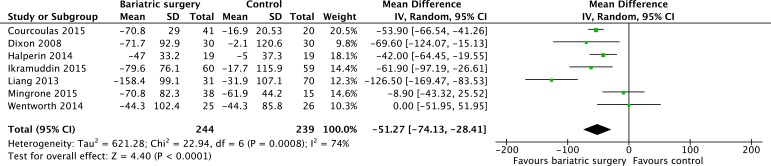
Mean changes in triglyceride concentrations (mg/dL) after bariatric surgery versus non-surgical treatment (control) for Type 2 diabetes mellitus (A conversion factor of 88.55 used to convert values from mmol/L to mg/dL).

7 studies [12, 19–23, 25] reported the mean changes of triglyceride concentration (mg/dL). Heterogeneity was high (*P* = 0.0008, *I*^2^ = 74%) and a random effect model was used. Bariatric surgery acquired more decrease of triglycerides compared with non-surgical treatment for T2DM (MD = -51.27, 95%CI: -74.13 to -28.41, *P* < 0.0001) (Figure 6).

### Meta-analysis of long-term outcomes

With respect to long-term outcomes, three endpoints including T2DM remission, quality of life, and adverse events were taken into analysis.

### Diabetes remission

**Figure 7:**
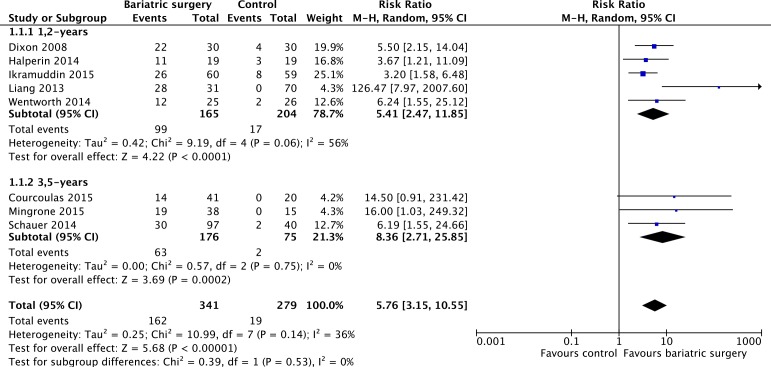
Type 2 diabetes remission after bariatric surgery versus non-surgical treatment (control) for Type 2 diabetes mellitus Subgroup analysis was done for the 1,2-year follow-up studies versus 3,5-year follow-up studies.

T2DM remission rate was the primary end point. All of 8 studies [12, 19–25] reported the relative risk of T2DM remission. Bariatric surgery acquired higher diabetes remission compared with non-surgical treatment for T2DM (RR = 5.76; 95%CI: 3.15 to 10.55, *P* < 0.00001). Analysis indicated that there was moderate heterogeneity among the studies (*P* = 0.14, *I*^2^ = 36%) and a fixed effect model was used. Further subgroup analysis showed that T2DM remission was no significantly different between the 1,2-year follow-up studies and the 3,5-year follow-up studies (*P* = 0.53, *I*^2^ = 0%) (Figure 7).

### Quality of life

There were 3 studies [20, 23, 24] reported quality of life which was assessed by the 36-Item Short Form Health Survey (SF-36). Schauer 2014 reported that five of eight mental and physical domains improved more among patients in the gastric-bypass group and two of eight domains in the sleeve-gastrectomy group, as compared with the medical-therapy group [24]. Halperin 2014 reported that SF-36 total scores were no significantly difference between bariatric surgery group and non-surgery group in at 1 year [20]. Mingrone 2015 reported that surgical patients scored significantly better than medically treated patients for all subdomains of quality of life and for the total score domains [23].

### Adverse events

**Figure 8:**
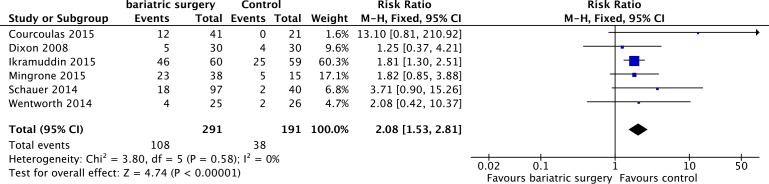
Adverse events after bariatric surgery *versus* non-surgical treatment (control) for Type 2 diabetes mellitus

There were 6 studies [12, 19, 21, 23–25] reported the adverse events. There was no heterogeneity (*P* = 0.58, *I*^2^ = 0%) among the studies, so a random effect model was used. Our outcome was prone to a higher adverse events rate in bariatric surgery compared with non-surgical treatment for T2DM (RR = 2.08, 95%CI: 1.53 to 2.81, *P* < 0.00001) (Figure 8). However, early surgical complications were relatively benign. There were no perioperative deaths and no late surgical complications in bariatric surgical groups after 1-year follow-up. The only 5-year follow-up study reported that five major complications of diabetes (including one fatal myocardial infarction) arose in four (27%) patients in the medical group compared with only one complication in the bariatric surgery groups. [23]

### Publication Bias

The publication bias in this meta-analysis was assessed by funnel plots to evaluate the reliability. However, the results of these tests are not reported, because this method is known to be unreliable when there are fewer than 10 studies in the meta-analysis [27] and because our qualitative analysis indicated a high likelihood of reporting bias.

## DISCUSSION

Bariatric surgery is used at first to treat obesity that is often associated with T2DM. Some studies found that obesity patient could obtain T2DM remission after bariatric surgery. Further research indicated that mild to moderate obesity patients could also obtain T2DM remission after bariatric surgery. Therefore, this meta-analysis is to investigate the effective of bariatric surgery treatment for T2DM with or without obesity. It remains to be established whether bariatric surgery is an effective treatment for T2DM and how bariatric surgery compares with non-surgical treatment with respect to short-and long-term outcomes [18, 28]. And recent initial studies [19, 21, 23–25] have reported the feasibility, safety, and efficacy of bariatric surgery for T2DM. However, the studies are limited to a small number of patients. There is no convincing evidence on the efficacy of bariatric surgery for T2DM. Our goal of this meta-analysis was to evaluate the efficacy of bariatric surgery for T2DM compared with non-surgical treatment. We comprehensively identified all relevant RCTs and summarized the evidence on short- and long-term outcomes.

Diabetes remission is the most important question regarding the use of bariatric surgery for T2DM. Our analysis demonstrates that overall T2DM remission of bariatric surgery compared with non-surgical treatment were significantly different, 47.5 vs 6.8% (*P* < 0.001), respectively. And then, the efficacy of T2DM remission was quantified from the pooled risk ratios based on the 8 RCTs. Our pooled outcome provided convincing evidence that bariatric surgery led to higher T2DM remission than non-surgical treatment for up to 5-year follow-up.

With respect to short-term outcomes, bariatric surgery acquired more reduction HbA_1c_, FPG, BMI, weight, and triglyceride, more increase HDL cholesterol. However, changes of LDL and total cholesterol were no significantly different. Moreover, there were no perioperative deaths and no late surgical complications in bariatric surgical groups. This meta-analysis demonstrates that bariatric surgery for T2DM is more efficient than non-surgical treatment.

Three studies [19, 23, 24] in this meta-analysis proved that bariatric surgery compared with non-surgical treatment in glycemic control is not only marked improvement in short term, but also more durable over the long term. Schauer et al. reported a 31% rate of T2DM remission maintenance in bariatric surgery patients at 3-year follow-up vs 5.0 % in the medical therapy group [24]. Similarly, Courcoulas et al. reported a 34.1% rate of T2DM remission at 3-year follow-up vs none of intensive lifestyle weight loss intervention participants [19]. In addition, Mingrone et al. recently reported 50% of the surgical patients maintained diabetes remission at 5-year follow-up vs none of the medically treated patients [23].

The reduction of long-term diabetes-related complications is an important aspect regarding the use of bariatric surgery for the treatment of T2DM. A 5 year follow-up RCT shows that medically treated patients are greater incidence of diabetes-related complications including a fatal myocardial infarction and bariatric surgery could reduce risk of diabetes complications [23]. This finding is supported by a very long-term follow-up observational study showing that bariatric surgery is associated with fewer microvascular and macrovascular complications than usual care [29].

Our results are supported by two systematic reviews. A review and meta-analysis from 2013 [30] included three randomized controlled trials. It showed greater weight loss, glycemic control, and diabetes remission during 1 to 2 years of follow-up after bariatric surgery compared with non-surgical treatment. The other review and meta-analysis from 2014 [28] included 5 RCT studies and 11 observational studies and had similar results.

There are several limitations in this meta-analysis. Although the number of available RCTs was noteworthy, the evidence is insufficient to reach conclusions that bariatric surgery is the preferred treatment for T2DM than non-surgery treatment. Most of included studies were relatively small sample sizes and lack of long-term results. Only 3 studies had follow-up longer than 3 years [19, 23, 24]. Additionally, many of the studies were from single a single site, which may affect generalizability. A greater number of larger and even more definitive studies would have increased this study's predictive strength.

Another limitation was the diversity of T2DM diagnosis and remission standards reported. Many studies determined T2DM outcomes idiosyncratically; the majority of studies did not define T2DM remission uniformly, some employing American Diabetes Association criteria, and others only biochemical marker of glycemic control. A uniform standard for reporting T2DM remission is needed to improve the scientific evidence base and support clinical decision making.

Another potential limitation was the intensity of patients treated by bariatric surgery may be inherently greater. Intensity of treatment in surgical therapy may improve weight loss and glucose outcomes. Caution is warranted when considering differences between surgical and non-surgical treatment when the intensity of treatment is not balanced in the two groups. Future studies should pay careful attention to this potential confounding factor.

In conclusion, our meta-analysis summarizes the best available evidence for short- and long-term results in directly comparative research studies of bariatric surgery vs non-surgical treatment in T2DM patients. This study demonstrates that bariatric surgery is associated with greater improvements in T2DM remission, HbA_1c_ and fasting plasma glucose levels, weight loss, and hyperlipidemia than non-surgical treatment such as medications, diet, and behavioral changes for patients with T2DM. However, results are limited to a small number of studies and individuals and lack data about the durability of benefit. To fully characterize the efficacy of bariatric surgery for T2DM, the evidence calls for further research on larger, well design, and long-term outcomes studies.

## MATERIALS AND METHODS

### Eligibility criteria

Studies were included for this meta-analysis if they met the following criteria: 1) study design: randomized controlled trials and each study to have at least 1-year follow-up; 2) investigated currently used laparoscopic or open bariatric surgery techniques (Roux-en-Y gastric bypass, adjustable gastric banding, sleeve gastrectomy, or biliopancreatic diversion); investigated as comparator non-surgical treatment for T2DM (diet, weight reducing drugs, behavioral therapy, or any combination thereof); 3) results: description of the details of T2DM remission, metabolic outcomes (glucose, lipids), weight loss, quality of life, and adverse events. The exclusion criteria included: 1) abstracts, letters, editorials and expert opinions, reviews without original data, case reports; 2) studies not reporting clinical outcomes of effectiveness or adverse events; and 3) studies with a sample size fewer than 10.

### Information sources and search strategy

We performed a comprehensive search in PubMed, Embase and Cochrane Library databases to identify all relevant studies available from their inception to June 15th 2016. We also searched trial registries of ongoing trials.

The search strategy followed the identification and screening guidelines established by the Preferred Reporting Items for Systematic Reviews and Meta-Analyses (PRISMA) statement. The search terms included (“diabetes or diabetic*” or “diabetes mellitus”) and (“bariatric surgery” or “metabolic surgery” or “diabetes surgery” or “gastric banding” or “sleeve gastrectomy” or “gastric bypass” or “duodenal switch” or “biliopancreatic diversion”) and (randomized controlled trial). These terms were used in different Boolean combinations. Limits set to govern the searches stipulated journal articles on adult humans written in the English language. All eligible studies were retrieved, and the reference lists of the identified studies and reviews were evaluated or additional studies.

### Data extraction

Two review authors (X.S. and X.F.) independently extracted data, and screened the quality and content of the included studies. Variable data of interest were extracted from included studies and entered into a dedicated database. The following data was extracted from each study: study characteristics (bariatric procedures, conventional treatments, study designs, bariatric surgery techniques, non-surgical treatments, and follow-up time), demographic and anthropometric measures (publication year, country, age, gender, BMI), T2DM remission, glycemic control (HbA_1C_ and FPG), weight loss, lipids, quality of life, and adverse events. The other authors (Y.F. and Z.F.) checked for data accuracy and completeness. Discrepancies were resolved by consensus in all authors.

### Risk of bias assessment

The quality of the inclusion of trials was assessed using the Cochrane risk of bias tool [26]. Two reviewers (G.W. and H.Z.) independently assessed risk of bias of the inclusion of studies. Disagreement was resolved by consensus.

### Data synthesis and statistical analysis

All data were analyzed using Review Manager version 5.3 for Mac. RR along with 95% CI was calculated for dichotomous data, and MD along with 95% CI was calculated for continuous data. The mean change of the included studies from baseline to end of follow-up was calculated. Missing standard deviations were derived from other statistics, such as *P* values or confidence intervals if needed [31]. For example, *P* = 0.00001 was assumed when a *P* value was reported as *P* < 0.00001. When only a range was reported, a formula was used to estimate Standard deviation (SD): Estimate SD = Range/4 (15 < n < 70); Range/6 (n > 70), and median was approximately equal to mean [32]. Heterogeneity among combined study results was assessed by Cochran's Q test and by the degree of inconsistency (*I*^2^). A random effect model was used if *P* < 0.05 and *I*^2^ > 50%. Otherwise, data were pooled by using the fixed effect model [27]. In the integration results, *P* < 0.05 indicated statistical significance. Publication bias in outcomes was assessed and treated using standard methodology. Publication bias was analyzed using a funnel plot.
